# Photoelectrocatalytic Surfactant Pollutant Degradation and Simultaneous Green Hydrogen Generation

**DOI:** 10.1021/acs.iecr.3c00840

**Published:** 2023-06-02

**Authors:** Katherine Rebecca Davies, Michael G. Allan, Sanjay Nagarajan, Rachel Townsend, Vijayshankar Asokan, Trystan Watson, A. Ruth Godfrey, M. Mercedes Maroto-Valer, Moritz F. Kuehnel, Sudhagar Pitchaimuthu

**Affiliations:** † SPECIFIC, Faculty of Science and Engineering, Swansea University, Swansea SA2 8PP, Wales; ‡ Department of Chemistry, Faculty of Science and Engineering, Swansea University, Singleton Park, SA2 8PP Swansea, Wales; § Department of Chemical Engineering, University of Bath, Bath BA2 7AY, U.K.; ∥ Swansea University Medical School, Faculty of Medicine, Health and Life Science, Singleton Park, Swansea University, Swansea SA2 8PP, U.K.; ⊥ Environmental Inorganic Chemistry, Department of Chemistry and Chemical Engineering, Chalmers University of Technology, Kemivägen 10, S-412 96 Göthenburg, Sweden; # Research Centre for Carbon Solutions (RCCS), Institute of Mechanical, Processing and Energy Engineering, School of Engineering and Physical Sciences, Heriot-Watt University, Edinburgh EH14 4AS, U.K.; g Fraunhofer Institute for Wind Energy Systems IWES, Am Haupttor 4310, 06237 Leuna, Germany

## Abstract

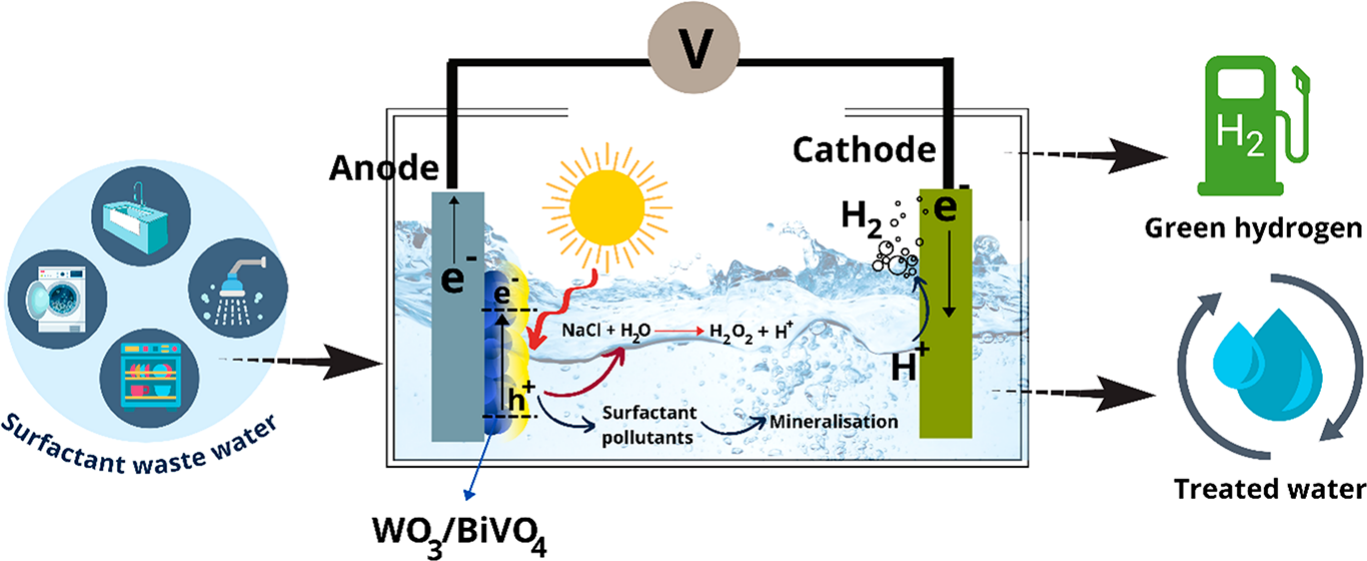

For the first time, we demonstrate a photoelectrocatalysis technique for simultaneous surfactant pollutant degradation and green hydrogen generation using mesoporous WO_3_/BiVO_4_ photoanode under simulated sunlight irradiation. The materials properties such as morphology, crystallite structure, chemical environment, optical absorbance, and bandgap energy of the WO_3_/BiVO_4_ films are examined and discussed. We have tested the anionic type (sodium 2-naphthalenesulfonate (S2NS)) and cationic type surfactants (benzyl alkyl dimethylammonium compounds (BAC-C12)) as model pollutants. A complete removal of S2NS and BAC-C12 surfactants at 60 and 90 min, respectively, by applying 1.75 V applied potential vs RHE to the circuit, under 1 sun was achieved. An interesting competitive phenomenon for photohole utilization was observed between surfactants and adsorbed water. This led to the formation of H_2_O_2_ from water alongside surfactant degradation (anode) and hydrogen evolution (cathode). No byproducts were observed after the direct photohole mediated degradation of surfactants, implying its advantage over other AOPs and biological processes. In the cathode compartment, 82.51 μmol/cm^2^ and 71.81 μmol/cm^2^ of hydrogen gas were generated during the BAC-C12 and S2NS surfactant degradation process, respectively, at 1.75 V RHE applied potential.

## Introduction

Surfactants are amphiphilic molecules consisting of both hydrophobic and hydrophilic parts^1^ and are commonly found in various daily domestic products such as soaps, shampoo, toothpaste, disinfectant sprays, laundry detergents, fabric conditioner, etc. They are also used in a number of industries such as leather, pharmaceutical, textile, paint, food, mining, paper and pulp, and pesticide for emulsification, solubilization, sterilization, and other applications.^2−4^ There are several types of surfactants which are classified based on the charge they carry on their hydrophilic head: nonionic (no charge), anionic (negatively charged head), cationic (positively charged head), or amphoteric (both positively and negatively charged head).^1,2^ The surfactant tail is often hydrophobic. Although surfactants are nontoxic at low concentrations, they have a lasting negative impact on aquatic ecosystem and public health.^5,6^

Most surfactants could be partially degraded during the wastewater treatment process, leading to the formation of more toxic intermediate byproducts that can still persist in surface waters, soils, and sediments.^4^ Among these most toxic surfactants are a class of cationic compounds called the quaternary ammonium compounds (QACs) that are antibacterial in nature and used commonly in disinfectants and detergents.^2^ The increased use of QAC has resulted in high concentrations (500 μg/L) entering wastewater.^2,7^ Incomplete degradation and partial removal of QACs have led to its concentrations in the range of 40–50 μg/L in surface waters.^7^ However, due to their chemical nature, QACs have a higher tendency to adsorb to soils and sediments leading to a significantly higher concentration of ∼9 g/kg sludge.^8^ It is highly concerning as excessive exposure of QACs to microbes could raise microbial resistance to these compounds. Hence, it is essential to remove these compounds from the water completely. Benzyl alkyl dimethylammonium compounds (BACs) with 12–16 carbon units^9^ are the most frequently detected QACs in wastewater.^7^ A 12 carbon unit BAC (BAC-C12) was used as one of the model compounds and its photoelectrocatalytic degradation was investigated in this work.

Anionic surfactants are another commonly used class of surfactants.^2^ They are widely utilized in detergents, cosmetics, and soaps.^2^ One of the most common types of anionic surfactants is linear alkylbenzenesulfonates (LASs).^10^ It has been evidenced that LASs are biodegradable in wastewater treatment plants under aerobic conditions.^2,10^ However, these compounds still occur in surface waters as current wastewater treatment technologies are inadequate to completely remove LASs from water. LAS effluent and sludge concentrations of >1000 μg/L and >30g/kg dry sludge, respectively, have been reported which are concerning.^4^ The other challenge of biodegrading LASs is that they can alter the microorganisms, thus hindering their performance for other pollutants.^2^ The problem with LASs entering surface water is that they are toxic to a range of aquatic organisms, such as invertebrates.^11^ Several methods have been reported on the degradation of LAS compounds, including physical and biological routes. While biological routes can mediate partial (at times complete) degradation, physical removal routes separate and transfer the surfactants from one medium to other in a concentrated form (e.g., water to sediment). Therefore, it is necessary to develop an alternative, sustainable technology that can completely degrade LAS compounds from wastewater. Sodium 2-naphthalenesulfonate (S2NS), a LAS was used as a model anionic surfactant, and its photoelectrocatalytic degradation was investigated in this work.

Among the existing methods for surfactant degradation, advanced oxidation processes (AOPs) have shown promising results for its complete removal.^12−17^ For instance, light-driven photocatalysis was reported to degrade 86% of LAS from wastewater in 60 min,^18^ however, inadequate charge carrier separation at the semiconductor photocatalyst/electrolyte interface led to a slower LAS degradation rate.^18^ This issue can be overcome by implementing a photoelectrocatalytic (PEC) process where a small applied potential to the circuit can enhance charge separation at the semiconductor/electrolyte interface to enhance the surfactant degradation rates. PEC has already been proven to degrade many persistent organic pollutants by producing highly oxidizing species such as hydroxyl radicals, active chlorine, hydrogen peroxide, etc. Importantly, from a scaleup point of view, catalyst particle separation is not required.

Surfactant degradation is an oxidation reaction that occurs at the (photo)anode. By virtue of the PEC process, a reduction reaction has to therefore occur (at the cathode). By carefully optimizing the reaction parameters, it is possible to obtain desired products at the cathode, alongside anodic surfactant degradation. When the electrodes are compartmentalized via appropriate proton/cation exchange membranes, product separation can be enabled. This is however complicated in the case of other AOPs such as photocatalysis as the reaction often occurs in a single reactor. Simultaneous hydrogen generation (at the cathode) alongside surfactant degradation (anode) was therefore investigated in this work.

Due to the growing interest in renewable green hydrogen as a low carbon energy vector, global focus has significantly shifted toward water electrolysis. Water electrolysis can only remain green when renewable electricity (solar/wind based electricity) is used. In addition, the high freshwater footprint of 9 kg water/1 kg hydrogen produced does not make water electrolysis a feasible and sustainable hydrogen producing technology. To overcome this, wastes can be utilized as feedstock (instead of water) for green hydrogen generation. PEC enables the use of waste (surfactant pollutant in wastewater) as the main feedstock, extracts electrons from the surfactants, degrades the pollutant, and simultaneously produces green hydrogen.^19,20^ With the surfactants acting as the sacrificial electron donor and the PEC process mediating effective charge carrier separation, this simultaneous process is promising. With the appropriate choice of photoanodes, a range of pollutants could be degraded while simultaneously generating green hydrogen at the cathode. A number of semiconductors such as TiO_2_, WO_3_, CdS, BiVO_4_, C_3_N_4_, Ta_2_O5, etc., have been reported as photoelectrodes for water oxidation and pollutants degradation.^19−24^ More novel materials are also being reported recently with good short-term but unstable performances due to the photocorrosion issue.^25,26^ Furthermore, semiconducting heterostructured nanocomposites also encourage solar-driven PEC reactions.^27−30^ The WO_3_/BiVO_4_ heterostructure photoanode reported here is however a promising candidate for oxidation due to its high charge separation,^31^ visible light activity, and suitable band positions for producing effective oxidants (free radicals, hydrogen peroxide, etc.).^32−35^ This work presented here is the first-of-its type and aims to investigate the simultaneous surfactant pollutant degradation and green hydrogen generation using mesoporous WO_3_/BiVO_4_ electrodes as photoanode in a two compartment PEC cell.

## Experimental Section

### Materials

All the chemicals were received from Sigma-Aldrich unless stated otherwise. WO_3_ nanocrystalline powder (100 nm size), fluorine-doped tin oxide coated (FTO) glass (Pilkington 12 Ω sheet resistance), α-terpineol, ethyl cellulose, ammonium metavanadate, bismuth nitrate pentahydrate, citric acid, nitric acid, ethanol, benzyldimethyldodecylammonium chloride, sodium 2-naphthalenesulfonate.

### Photoanode Fabrication

In the first step, WO_3_ paste was prepared using commercial WO_3_ powder (Sigma-Aldrich). Further, the paste is coated onto a well cleaned FTO substrate. Following that, a BiVO_4_ thin film was coated on the WO_3_ layer using spin coating technique. Finally the WO_3_/BiVO_4_ coated on FTO substrate is calcinated at 450 °C for 30 min. A detailed experimental procedure of WO_3_/BiVO_4_ photoanode is explained in the Supporting Information (S1). Note that the optimized BiVO_4_ films were coated based on the previous report.^31^ The thicknesses of the WO_3_ and BiVO_4_ films play a critical role in PEC performance. Therefore, we have optimized the thicknesses of each layer to obtain higher photocurrent density in the PEC cell. Finally, all experiments use the 2 layers of WO_3_ coating and 1 layer of BiVO_4_ coating as benchmarking photoanode configuration.

### Characterization

The X-ray diffraction measurements were conducted utilizing a Bruker D8 Discover X-ray diffractometer with a copper source (40 kV, 40 mA) and a 1D detector in Bragg–Brentano. The scanning electron microscopy (SEM) images were captured using a JEOL 7800F FEG-SEM with an Oxford Instrument X-MaxN energy dispersion spectra (EDS) detector with a 50 mm^2^ window. The high resolution-transmission electron microscopy (HR-TEM) image was captured using a FEI Technai F2O system. The X-photoelectron spectroscopy (XPS) measurements were achieved by utilizing a Kratos Axis Supra using a monochromatic Al Kα X-ray source operated at 225 W (15 mA emission current). The UV–vis diffuse reflectance spectra (UV–vis DRS) were obtained using a PerkinElmer Lambda 365 system.

### Photoelectrocatalysis Experiments

PEC experiments were carried out using a two-compartment cell. The anodic and cathodic compartments were separated with a proton exchange membrane (Nafion 117). The WO_3_/BiVO_4_ photoanode is applied as a working electrode, platinum wire is used as a cathode, and 0.5 M aqueous NaCl is used as electrolyte. The Ag/AgCl reference electrode is used for all experiments. The chronoamperometry experiments are demonstrated with a Zahner potentiostat (Thales Zahner Zennnium) at an applied potential of 1.2 V (vs Ag/AgCl) to the WO_3_/BiVO_4_ working electrode and a 30 s off/on chopped light of 1 Sun solar light (Solar Simulator 350–1800 nm, HAL-320W, Asahi Spectra) for 5 min. The linear sweep voltammetry experiments were conducted by using a Zahner potentiostat (Thales Zahner Zennnium) with an applied potential range from −0.5 to 1.5 V (vs Ag/AgCl) and carried out in the dark and 1 Sun condition (Solar Simulator 350–1800 nm, HAL-320W, Asahi Spectra). All the experiments were repeated three times unless otherwise stated.

The applied potential values (vs Ag/AgCl) to RHE (NHE at pH = 0), is converted by the Nernst equation.
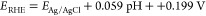
1where *E*_Ag/AgCl_ is the applied potential. The incident photon to current efficiency (IPCE) was measured using a xenon lamp (75 W, Hamamatsu) coupled with a monochromator (OBB-2001, Photon Technology International). The IPCE values are estimated using the procedure reported by Kafiza et al.^36^ For surfactants degradation experiments the BAC-C12 (50 μg/mL) and S2NS (50 μg/mL) were mixed individually in 1 M aqueous NaCl electrolyte and used for all experiments.

### Surfactants Analysis

To monitor and quantify the amount of surfactant (BAC-C12 and S2NS) present in the sample, a liquid chromatography system coupled with a UV detector (LC-UV, Agilent 1200 LC-UV system) and a Waters X-select C-18 column (2.1 mm × 100 mm) was utilized. Samples (1 mL) were collected every 30 min and filtered utilizing the filters. An injection volume of 5 μL was utilized with a flow rate of 0.250 mL/min with gradient mixing of 0.1% formic acid in water (mobile Phase A) and acetonitrile (mobile Phase B) as the mobile phases (75:25 to 0:100 over 16 min, with a column wash at 100% B for 7 min and recondition step for 1 min and re-equilibrate for 10 min).

### Hydrogen Peroxide Quantification

Quantofix H_2_O_2_ stripes were utilized to quantify the H_2_O_2_ being produced during the surfactants degradation experiment. These stripes can only give an approximation with detection range between 0.5 and 25 mg/L. The stripes were dipped into the electrolyte every 30 min and examined against the color chart to obtain the H_2_O_2_ concentration.

### PEC Hydrogen Generation Experiments

H_2_ generation experiments were conducted similar to surfactants degradation experiments, except the cathodic compartment had an inlet and outlet line to and from a gas chromatography (GC) system. A PalmSens EmStat 3+ potentiostat was used. The PEC cell was irradiated with a solar simulator (Thermo-Oriel) equipped with an AM 1.5G filter (Newport) at 1 sun; experiments were performed in triplicate. A Shimadzu Nexis GC-2030 was used to quantify the hydrogen gas generated. The run time of the method was 5 min, programmed with an autoinjection after 2 min and a backflush at 2.29 min to avoid moisture from being injected into the column. The gas samples were carried from the PEC cell headspace by the purge gas (nitrogen, BOC) via a mass flow controller (Bronkhorst) to the GC sample loop (Restek, 2 mL). From the determined H_2_ concentration in the purge gas and the purge gas flow rate, the H_2_ evolution was calculated assuming constant H_2_ production in between sampling points.

## Results and Discussion

### Structure, Morphology, and Chemical Environment of WO_3_/BiVO_4_

**Figure 1:**
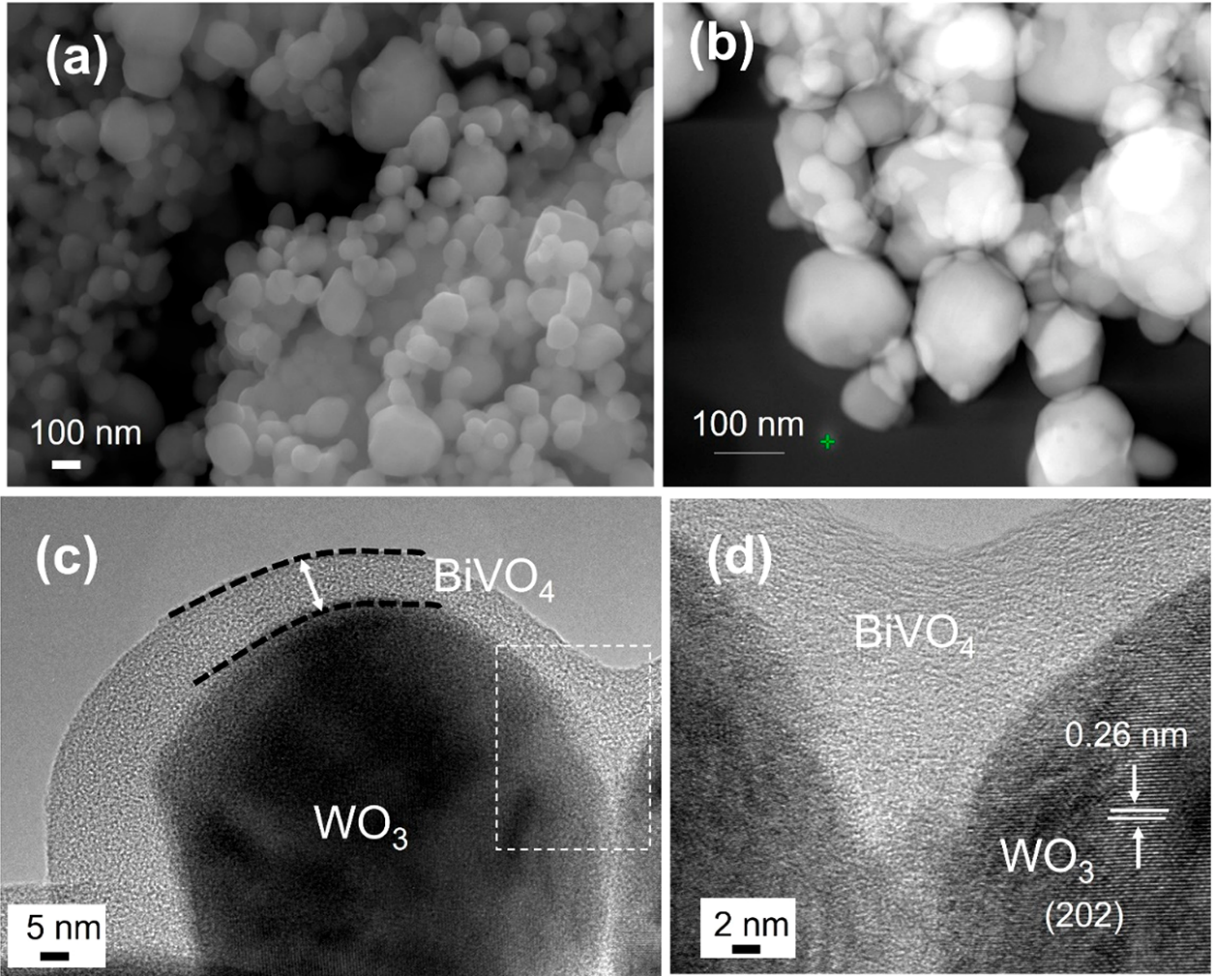
(a) SEM image of WO_3_/BiVO_4_ thin film coated on FTO substrate; (b) HAADF image of WO_3_/BiVO_4_ thin film; HRTEM images of WO_3_/BiVO_4_ thin film at (c) 5 nm and (d) 2 nm scale.

The crystal structure of the BiVO_4_ thin film coated on WO_3_ was examined with X-ray diffraction (XRD) spectra (Figure S1a,b). From Figure S1a the peaks exhibiting around 18.9° and 28.9° correspond to the (011) and (013) reflections of monoclinic scheelite BiVO4 (JCPDS 01-075-1866).^37^ The other predominant peaks are attributed to FTO substrate (indicated with asterisk (∗) symbol). When BiVO_4_ film is coated onto the WO_3_ films, it exhibits a weaker crystallite peak (Figure S1b) due to high crystalline nature of the WO_3_ film. The characteristic crystal structure of WO_3_ belonging to the orthorhombic structure was confirmed through the crystalline peaks seen at 23.1°, 23.6°, and 24.3°, which correspond to (002), (020), and (200) crystalline phases. A weak crystalline peak of BiVO_4_ was observed at 18.5° showing the suppression of the highly predominant BiVO_4_ crystallite peak by WO_3_ in the heterostructure. Further, the scanning electron microscope images were recorded to ensure the formation of BiVO_4_ thin film coating on the WO_3_ surface (Figure 1a). As seen, the WO_3_/BiVO_4_ particles were in a nonhomogeneous distribution and in the size range of 50–120 nm. The coating integrity of WO_3_/BiVO_4_ film onto the FTO substrate was examined with a cross-section (Figure S2a) and planar SEM image (Figure S2b) which showed uniform coating throughout the substrate. The elemental analysis spectra (EDS) presented in Figure S2c showed Bi, V, and O elements, confirming the presence of BiVO_4_.

**Figure 2:**
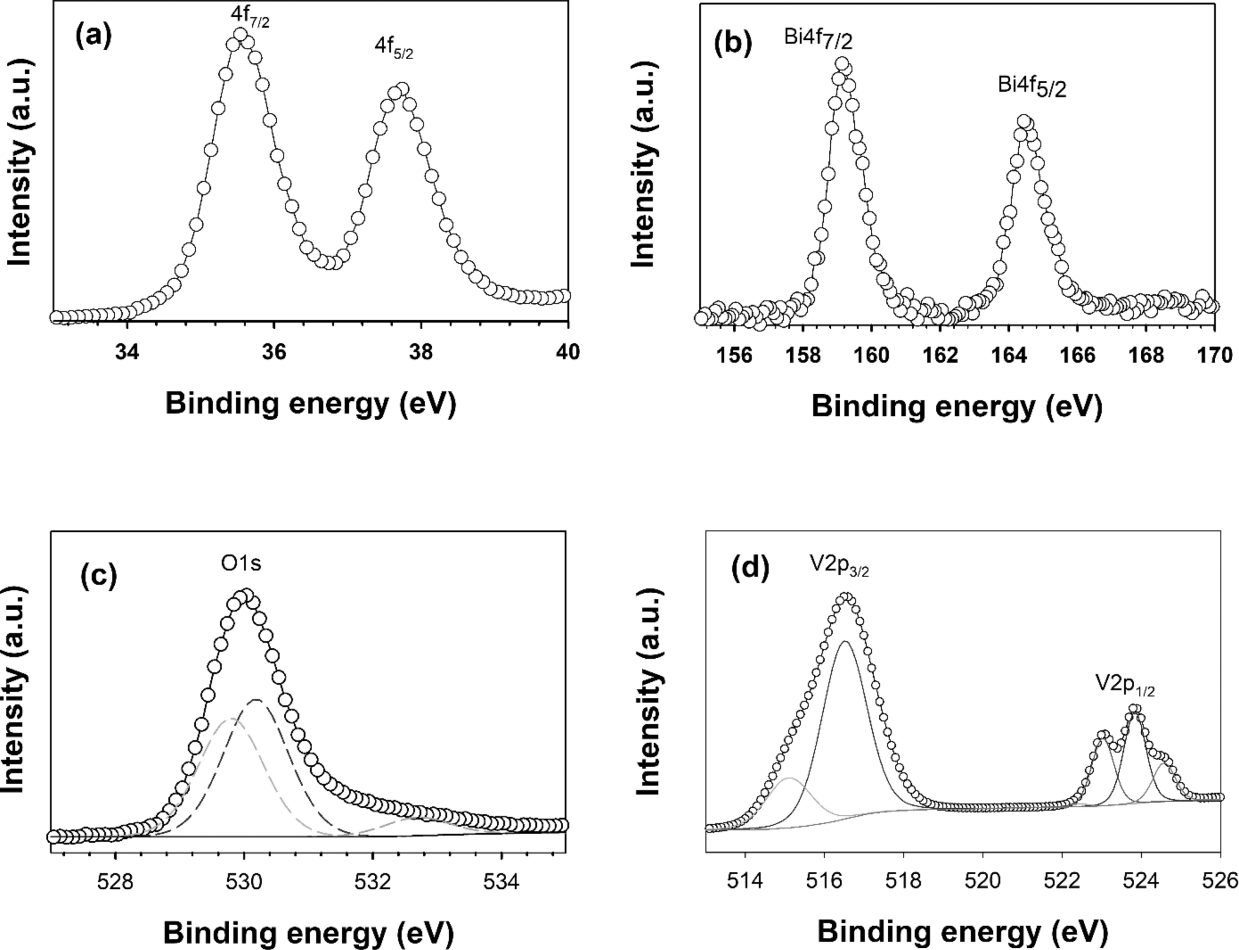
XPS results of WO_3_/BiVO_4_ thin film (a) W 4f, (b) O 1s, (c) Bi 4f, and (d) V 2p core spectra.

The high magnification HAADF image (Figure 1b) explored the proximity of BiVO_4_ thin film coated WO_3_ surface. The BiVO_4_ film randomly covered the WO_3_ surface, which differs from the conventional core–shell distribution decorated nanostructures.^38^ Unlike the vacuum-based coating technique, spin coating resulted in random crystal growth or thin film deposition on the target substrate or host semiconductor surface. However, a spin-coating method (1 cycle of BiVO_4_ coating) is an appropriate route compared with dip coating or drop-costing techniques to form an ideal interfacial structure to achieve intimate and uniform contact of the conformal BiVO_4_ layer on the WO_3_ surface.^39^ This heterojunction formation will be favorable for the charge carrier separation/transfer between BiVO_4_ and WO_3_. By further examining the spin-coated BiVO_4_ thin film on the WO_3_ particle surface at a 5 nm scale of TEM image (Figure 1c) we can see the heterostructure semiconductor junction formation. In the high magnification TEM image demonstrated at a 2 nm scale (Figure 2d), a distinct lattice fringe was observed with a diameter of 0.26 nm corresponding to the (021) phase of WO_3_. However, we could not obtain the crystal lattice plane information on BiVO_4_ as observed in the XRD results. The discrepancy between the XRD and TEM results are due to the measurement limitations of TEM. During TEM analysis, while examining the crystal lattice of BiVO_4_ at the different regions, the samples are often damaged. So, we could only get clear images of BiVO_4_ at the amorphous regions. Challenges in observing the crystal plane of thin metal oxide coatings by TEM have been reported by others.^40,41^

The chemical environment of WO_3_/BiVO_4_ film was examined with XPS. Figure 2a showed a strong peak at 35.55 and 37.66 eV, respectively, attributed to W 4f_7/2_ and W 4f_5/2_ orbitals ensuring the W^6+^ electronic state of WO_3_.^42−44^ The O 1s core spectra (Figure 2b) displayed the peak around 530.5 eV that can be split into multiple O 1s peaks associated with lattice oxygen (529.6 and 530.4 eV) and surface hydroxyl groups (−OH) (531.5 eV).^45^ The surface hydroxyl groups appeared at WO_3_/BiVO_4_ film, suggesting surface oxidation of the material. These −OH groups are complementary to enhance the water pollutant species attracted to WO_3_/BiVO_4_ film.^46^ Further analysis of the XPS spectra shows the peaks at 159.21 and 164.52 eV (Figure 2(c)) indicative of Bi 4f_7/2_ and Bi 4f_5/2_, respectively,^47^ and at 516.5 and 524.1 eV (Figure 2(d)) revealing V 2p_3/2_ and V 2p_5/2_, respectively,^43^ confirming that the target coordination states of Bi (3+) and V (5+) associated with BiVO_4_ have been formed within the electrode coating.

**Figure 3:**
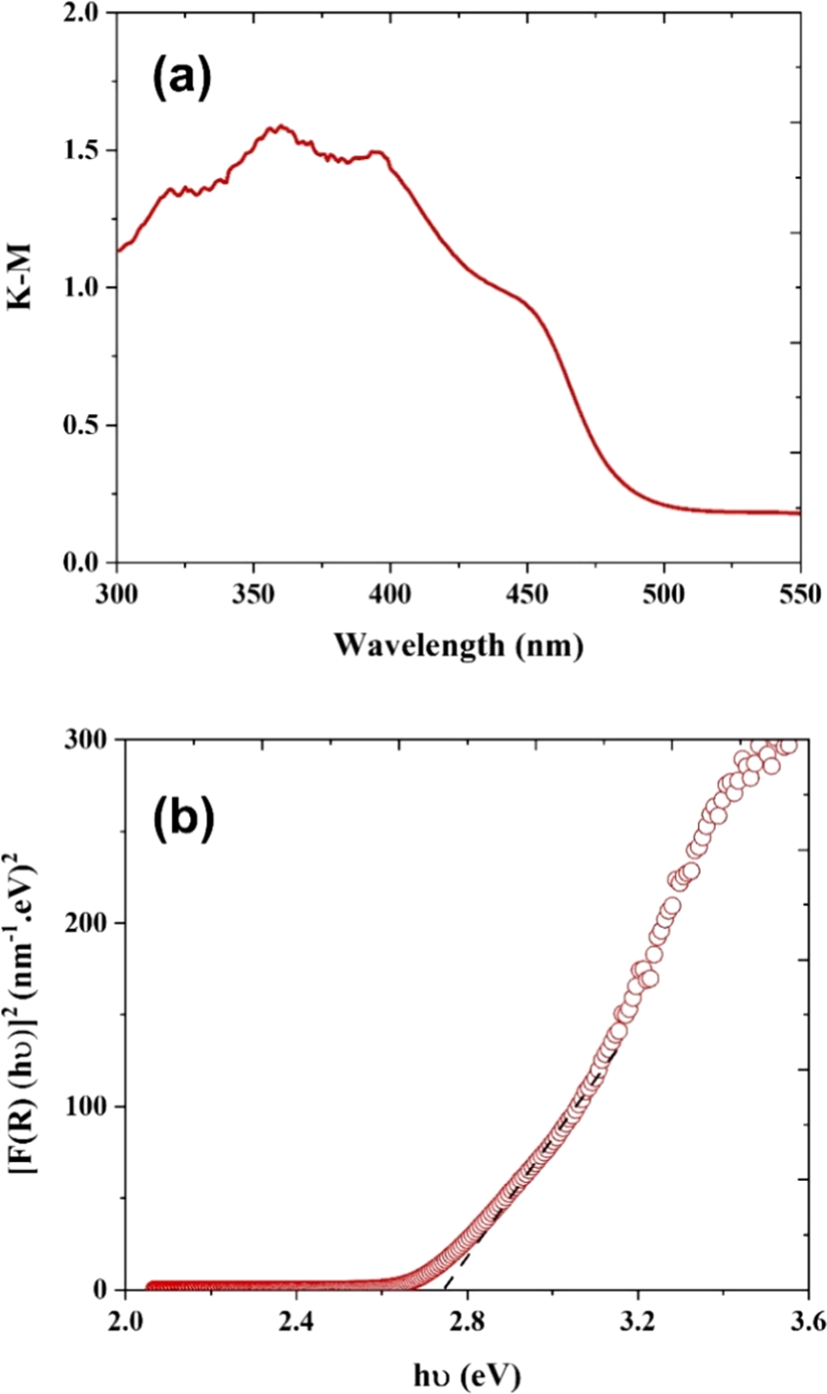
(a) Diffused reflectance spectra and (b) Kubelka–Munk plot of WO_3_/BiVO_4_ thin film coated on FTO substrate.

The optical absorbance property of the semiconductor is a crucial driver for enhancing the PEC process efficiency. The bandgap energy of the semiconductor dictates the corresponding wavelength of light that can be used for photoexcitation. The optical absorbance behavior of WO_3_, BiVO_4_, and WO_3_/BiVO_4_ film (Figure 3a) was examined using a spectrophotometer. The optical absorbance range of BiVO_4_ in the visible wavelength region is more extended than that of WO_3_ due to its bandgap characteristics (Figure S3).^23^ From Figure 3a, a broad light absorbance behavior was observed between 300 and 500 nm, indicating that WO_3_/BiVO_4_ film is an appropriate photoabsorber for harvesting visible light (e.g., natural solar light). The bandgap energy of the WO_3_/BiVO_4_ film is estimated using Kubelka–Munk plots (Figure 3b) using the relation^48^
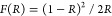
2where *R* corresponds to the diffuse reflectance. The estimated bandgap energy is found to be ∼2.76 eV.

### Photoelectrochemical Performance

#### Photoelectrochemical Water Splitting

**Figure 4:**
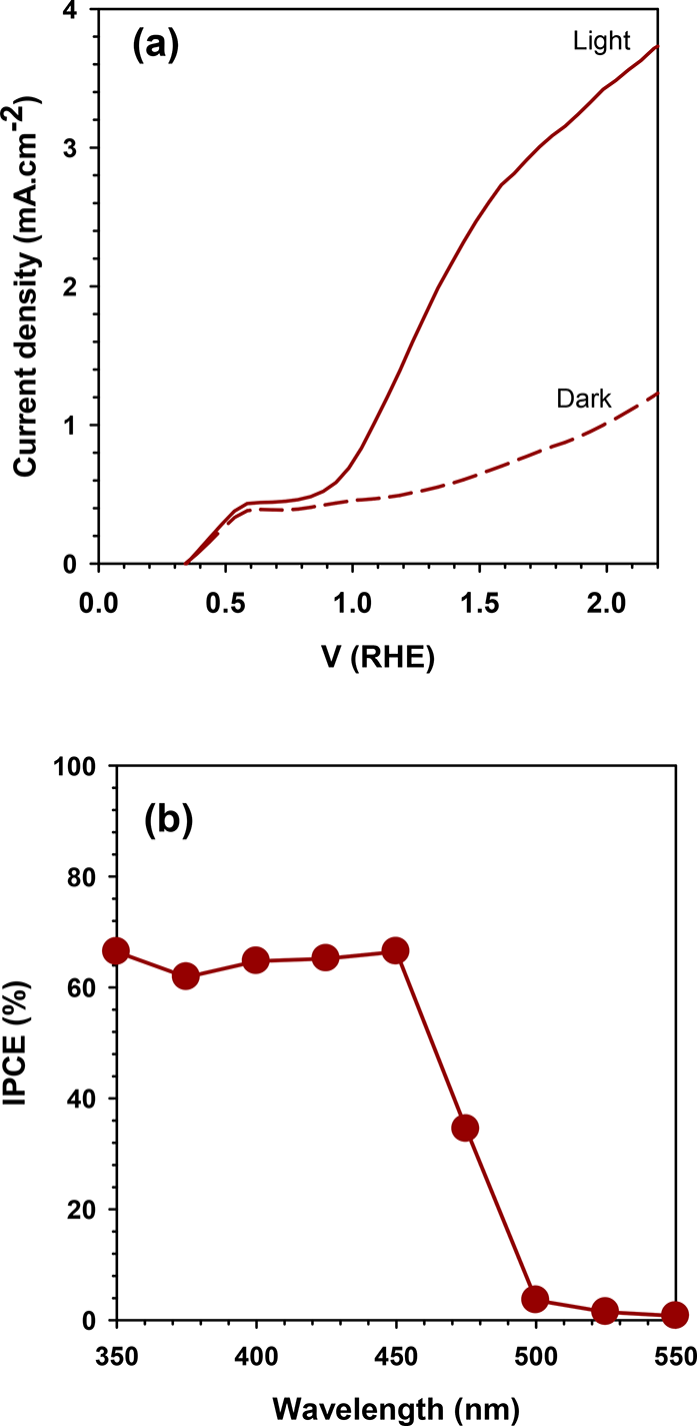
(a) JV plots and (b) IPCE results of WO_3_/BiVO_4_ photoanode in PEC water splitting reactions. Note that the 1 M NaCl solution is used as an electrolyte.

The photoelectrocatalytic performance of WO_3_/BiVO_4_ film was first examined for water splitting reactions. The *J*-*V* plot of the WO_3_/BiVO_4_ photoanode is presented in Figure 4(a). Under light irradiation, the WO_3_/BiVO_4_ photoanode starts generating the current in the circuit at above 0.35 V RHE applied potential. This potential refers to the on-set potential to remove the band bending at the WO_3_/BiVO_4_/electrolyte interfaces. Though a small current was observed at dark conditions due to current leakage from the substrate to the electrolyte, the photoanode still exhibits markedly higher current density ∼2.7 mA·cm^–2^ (after deducing dark current) upon light irradiation. It ensures the photoelectric effect at WO_3_/BiVO_4_. Briefly, the photoelectrons are excited from the valence band to the conduction band of semiconductors WO_3_/BiVO_4_ and are further injected into the charge collector (FTO) via an electron hopping mechanism. The photoholes, on the other hand, oxidize water molecules to oxygen gas. From Figure 4(a), the higher photocurrent observed at 2.2 V RHE applied potential indicates a higher water oxidation rate. Conversely, it also refers to a higher rate of proton reduction into hydrogen gas at the cathode. The quantum efficiency of the photoanode is examined at different wavelength regions. Figure 4b shows the IPCE results of the WO_3_/BiVO_4_ photoanode using 1 M NaCl aqueous electrolyte. From these results, it was understood that the photoanode was highly active between the 300 and 500 nm region, which reflects the optical absorbance results (as seen in Figure 3a). Overall, the WO_3_/BiVO_4_ photoanode was able to utilize 65% of light photons for water splitting reactions.

#### Photoelectrochemical Surfactant Degradation

**Figure 5:**
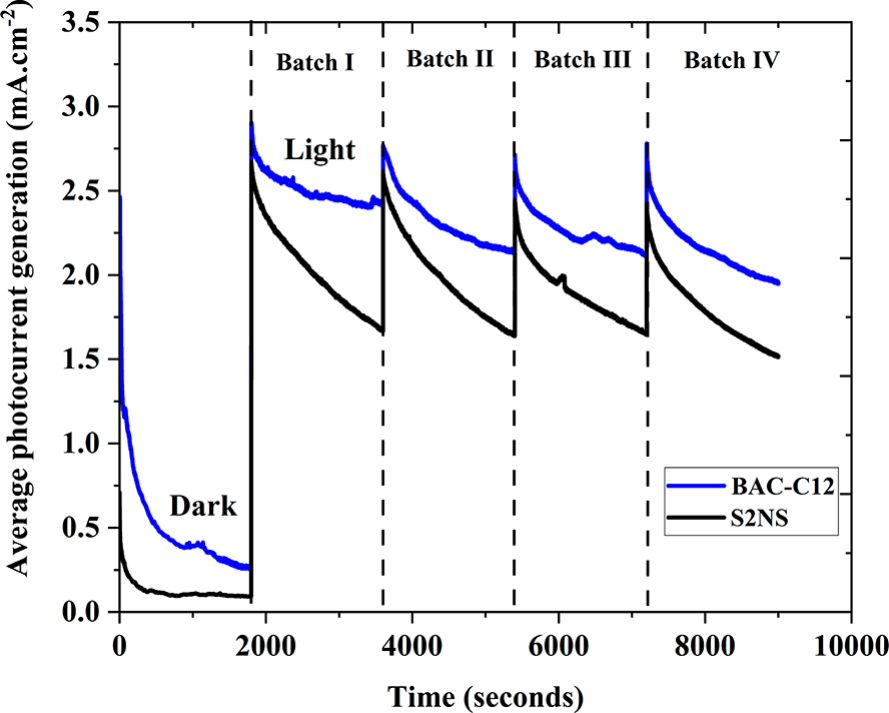
Chronoamperometry plot of WO_3_/BiVO_4_ photoanode with S2NS and BAC-C12 pollutants-based electrolyte. The experiment was recorded at 1.75 V RHE applied potential under dark and light conditions. The broken line represents the electrolyte and photoanode replacement period where the experiments were temporarily stopped for few minutes. Each experiment was carried out with fresh WO_3_/BiVO_4_ photoanode to evaluate the reproducibility of the WO_3_/BiVO_4_ photoanode (batch test) in PEC surfactant degradation.

The PEC degradation of BAC-C12 and S2NS has been monitored by chronoamperometry plots (Figure 5) displaying the current density produced in the circuit during the experiments. The experiments were run 4 times, and between each run the WO_3_/BiVO_4_ photoanode and electrolyte were replaced. The observed severe current decay from 2.7 to 1.6 mA/cm^2^ in each batch could be either due to surfactant degradation or photoanode corrosion.

To further understand this, the current densities observed in the presence and absence of surfactants were compared. It was inferred that there was no difference in photocurrent generation due to the presence of surfactants, i.e., the photocurrent generation was independent of the surfactants present in the electrolyte. Therefore, it was deduced that the photocurrent decay over time was due to photoanode instability (photocorrosion). It is well-known that the vanadium (V^5+^) species leaching from BiVO_4_ results in photocurrent reduction,^49,50^ which could be the case here. We have examined the elemental composition of W, Bi, O, and V of the photoanode before and after PEC process is examined (Table S1). It shows that a slight change in W, Vi, and O environment after PEC process may be due to the photocorrosion effect responsible for photocurrent reduction under a long period of operation.

**Figure 6:**
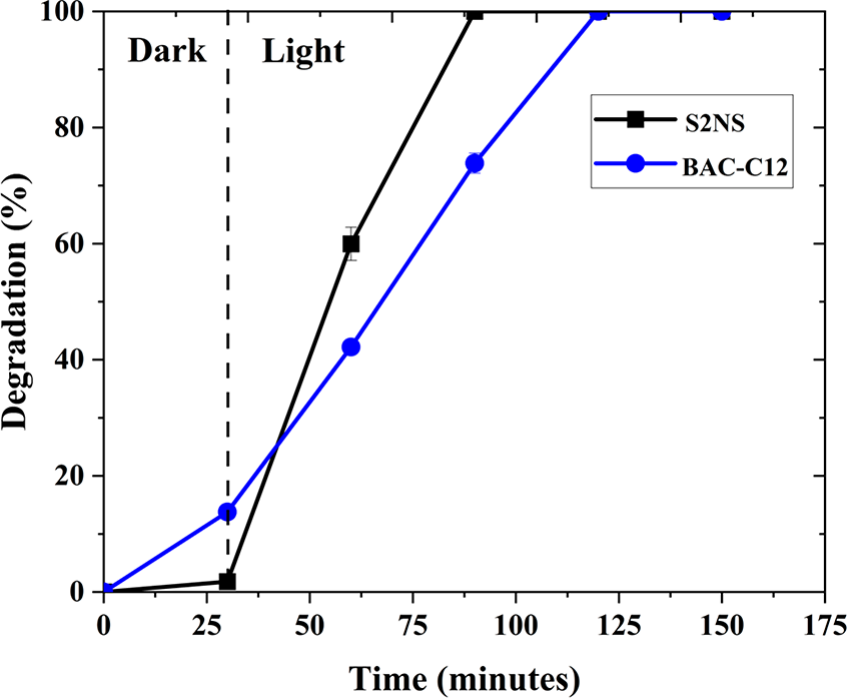
PEC surfactant degradation (%) at dark and light irradiation conditions. The experiment was recorded at 1.75 V RHE applied potential.

An identical pollutant concentration (50 μg/mL) of anionic surfactant S2NS and cationic BAC-C12 surfactants was tested in the PEC process. As seen in Figure 6, complete degradation of both the surfactants was achieved in this work, however, the rate of degradation of S2NS was higher compared to BAC-C12 (excluding the 30 min absorption equilibrium). After benchmarking, the representative chromatograms obtained for a pure BAC-C12 and S2NS standard are presented in Figures S4 and S5.

S2NS showed minimal dark adsorption onto the anode surface (Figure S6). This can be attributed to the chemical structure of the anionic surfactant. S2NS has a negative hydrophilic head, and the anode surface has hydroxyl ions that are negatively charged (as observed from the XPS results). This will lead to the surfactants repelling from the anode surface, leading to minimal dark adsorption. Upon irradiation of the photoanode, the electrons from the valence band migrate to the conduction band leaving behind positive holes. These positive holes migrate to the surface of the anode and can either oxidize water molecules or surfactants. In this case, due to the opposite charges, the surfactants have a higher affinity to the positive holes as opposed to water leading to a higher degradation rate (shorter time for complete removal). In the case of cationic surfactant (BAC-C12), higher surface adsorption to the anode was observed. The positively charged BAC-C12 has a strong affinity toward the negatively charged anode surface, leading to this higher adsorption (Figure 6). Upon irradiation, the positive holes oxidize water molecules predominantly, followed by surfactant degradation. Hence a slower degradation rate compared to anionic surfactants was observed. Interestingly, no byproducts were observed from mass spectra analysis. Therefore, it is evident that PEC-mediated remediation of surfactant-containing wastewater can be completely removed using a WO_3_/BiVO_4_ photoanode without the formation of hazardous byproducts as otherwise observed with some AOP-based degradation methods.

We have investigated the influence of initial concentration pollutants on byproduct formation and pollutant degradation rate. For instance, we utilized 100 μg/mL of BAC-C12, and small traces of unidentified byproducts were noticed (retention time of 15.46 min). However, when we reduced the concentration of BAC-C12 to 50 μg/mL, there was no byproduct peak noticed. The influence of initial concentration of BAC-C12 on PEC degradation rate was presented in Figure S7. It shows that 50 μg/mL concentration of BAC-C12 has a faster degradation than 100 μg/mL of BAC-C12 because byproduct formation is avoided. Therefore, the initial concentration of surfactant pollutants plays a critical role on degradation rate.

It is well recognized that the metal oxide-based photoanodes (TiO_2_, WO_3_, CeO_2_) in the PEC process can produce hydroxyl radicals (*OH) at 2.78 V vs NHE applied potential.^51−53^ These hydroxyl radicals have strong oxidizing properties, which can degrade pollutants in wastewater.^54^ However, no direct free radical generation is observed from the WO_3_/BiVO_4_ photoanode due to its valence band position being less than 2.8 eV (result not presented here). Alternatively, direct hole oxidation of surfactants was observed in this case. This is based on the commonly accepted phenomenon of Langmuir-Hinselwood-type photocatalytic processes.^55^ Additionally, the possibility of H_2_O_2_ generation during the PEC process was monitored. During the PEC process, H_2_O_2_ can be generated via a two-hole oxidation pathway as shown in the reaction in eq 3(35,56)
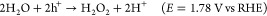
3(57)

**Figure 7:**
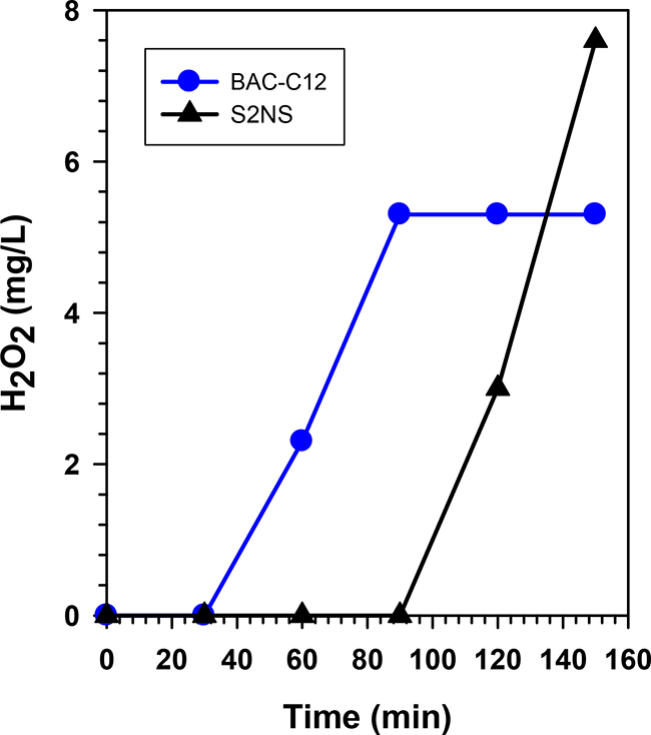
PEC anodic hydrogen peroxide generation in the presence of different surfactant pollutants (S2NS and BAC-C12).

The amount of H_2_O_2_ produced during the surfactant degradation experiments was determined using Quantofix H_2_O_2_ strips. These strips were utilized to give an approximation of H_2_O_2_ concentrations. Interestingly, a significant amount of H_2_O_2_ was generated from WO_3_/BiVO_4_ photoanode in the presence of surfactants. With the anionic surfactant S2NS, the negative charge possessed by the hydrophilic moiety has a higher affinity to positively charged holes. Hence the reactivity of holes with water is significantly reduced. In this case, with negligible competition for the valence band holes (until complete degradation of S2NS) no water oxidation products (H_2_O_2_) are observed. However, upon complete removal of S2NS, a steady linear increase in H_2_O_2_ concentration was observed (Figure 7). This further confirms that S2NS removal observed in this case was based on direct hole oxidation. With BAC-C12, the preadsorbed molecules during the dark phase were subjected to direct hole oxidation; however, due to similar charges on the photoholes and the surfactants, water oxidation is preferable over surfactant degradation. Hence an increase in H_2_O_2_ concentration (Figure 7) was observed alongside BAC-C12 degradation. It could be possible to argue that H_2_O_2_ would have influenced the degradation of these surfactants; however, this is unlikely. If this were to be the case, hydroxylated intermediates would have been observed in the mass spectra analysis. This therefore conclusively proves that the surfactants were degraded directly via the photogenerated holes and not indirectly via free radicals or H_2_O_2_. The production of H_2_O_2_ during pollutant degradation is of utmost importance when considering the degradation of real wastewaters. In such cases, while direct hole oxidation can facilitate surfactant degradation, the in situ generated H_2_O_2_ can mediate the degradation of coexisting pollutants in wastewater.

In addition to H_2_O_2_, other oxidants could also be formed in the PEC process when NaCl is used as the electrolyte. Photoelectrocatalytic oxidation of chloride ions could lead to the formation of active chlorine species such as Cl_2_, HClO, and Cl^–^ by the following reactions,^58,59^
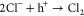
4
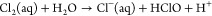
5
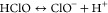
6

For instance, the amount of active chlorine produced during the PEC process varies with pH and temperature of the electrolyte. In a traditional water treatment process NaCl based solution will form Cl_2_ in acidic conditions (<pH 3.3), and HClO will be generated between pH 3.3–7.5. Furthermore, Zanoni et al.^58^ explored that PEC cells with NaCl electrolytes produce highly active chlorine at the lowest pH values. Therefore, the pH of the solution will indicate the type of active chlorine species generated from the NaCl electrolyte based PEC process. In the present work, the pH variation before and after PEC degradation of BAC-C12 was measured to be 5.80 to 2.60, and in the case of S2NS degradation, pH changed from 5.98 to 2.59. This indicates that active chlorine species could have been produced. However, to elucidate the role of these species in the degradation reaction, further work is required.

#### Simultaneous Hydrogen Generation

**Figure 8:**
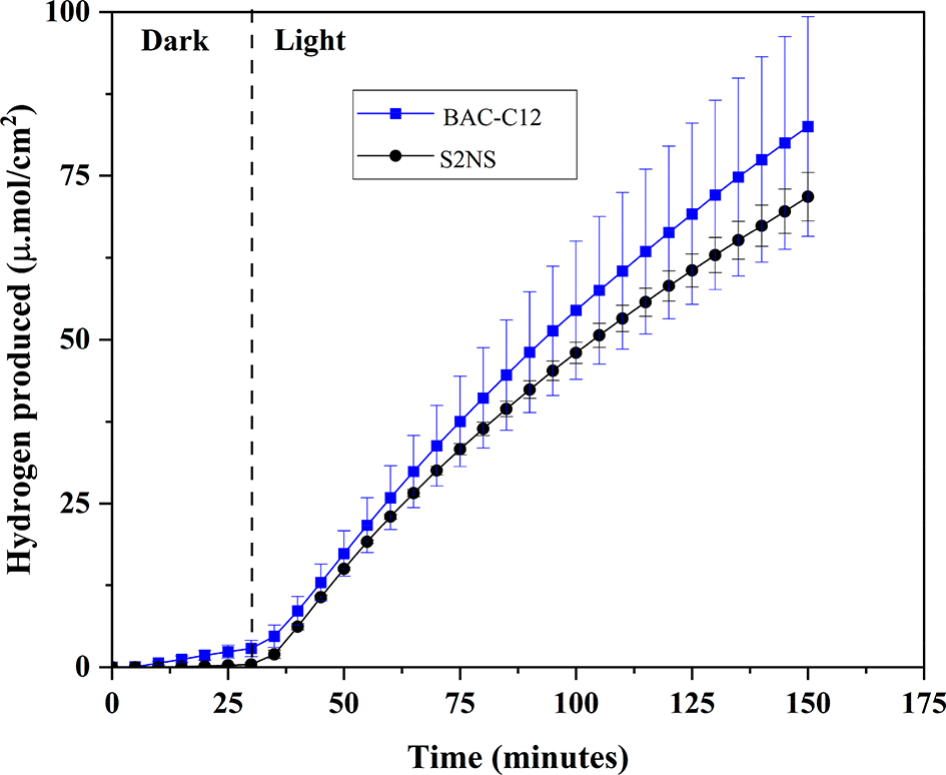
PEC hydrogen generation measured at cathode compartment during surfactants degradation process at the anode. This experiment was carried out at a two-compartment PEC cell at 1.75 V RHE applied potential under 1 sun.

While surfactant degradation was investigated at the photoanode, hydrogen gas evolution from the cathode occurred simultaneously. Similar to photocurrent being independent of surfactants present in the anode compartment, hydrogen evolution was also found to be independent of surfactants. The availability of protons from the water molecules influenced hydrogen evolution. This is conclusively shown in Figure 8 where a linear increase in hydrogen concentration was observed when the degradation of both the surfactants were investigated. Had the surfactants influenced hydrogen evolution, the gas generation profiles would have followed a similar trend to surfactant degradation and decayed over time. This means that injection of excess electrons (sacrificial electron donation) from surfactant oxidation did not occur. Zhao et al. on the other hand found that hydrogen generation from the pollutant degradation process depended on the removal efficiency.^60^ The higher was the removal efficiency, the higher was the hydrogen production, which indicated that a higher amount of photogenerated holes reacted with the pollutant. It meant the pollutant acted as a sacrificial agent,^60^ which is not the case here.

**Figure 9:**
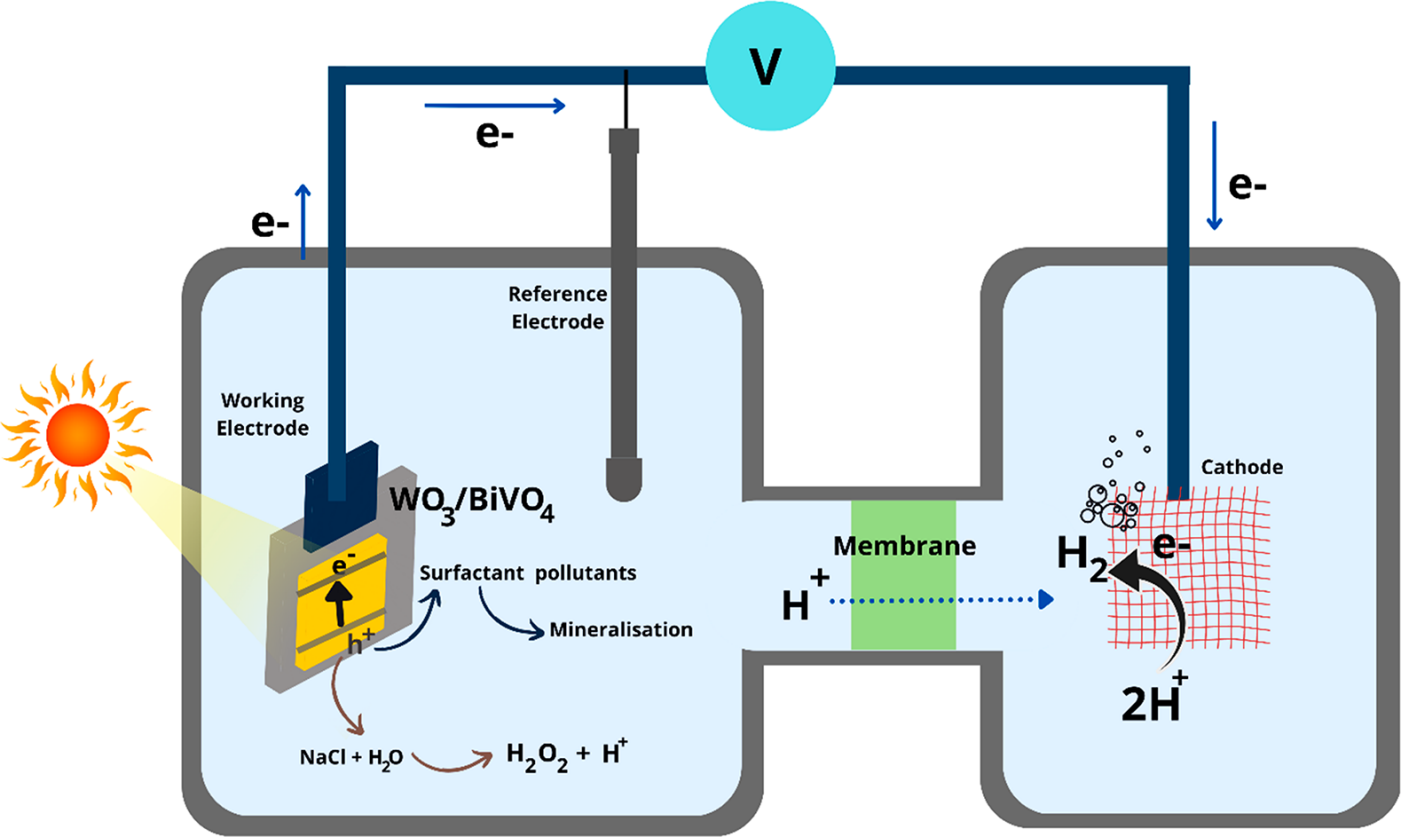
Schematic illustration of PEC cells for simultaneous surfactants degradation at the anode and hydrogen gas evolution at the cathode using a mesoporous WO_3_/BiVO_4_ photoanode.

Based on the investigation, a schematic illustration of surfactant degradation at the WO_3_/BiVO_4_ photoanode and hydrogen recovery at the cathode is shown in Figure 9. The photocharge carriers (e^–^ and h^+^) generated at WO_3_/BiVO_4_ heterojunction are separated, where the photoholes (h+) directly oxidize the surfactants alongside the production of hydrogen peroxide from competitive water oxidation. Protons (H^+^) generated at the anode are then transported to the cathode and reduced to hydrogen by photoelectrons generated by the photoanode. Both the surfactant’s degradation and hydrogen gas evolution are understood to be independent. This option offers to recover the clean hydrogen gas during wastewater treatment and avoid undesirable secondary reactions on the cathode.

## Conclusions

A mesoporous WO_3_/BiVO_4_ photoanode was successfully fabricated and tested for PEC water splitting reactions where it generated a current density of 2.7 mA·cm^–2^ at 1.75 V RHE applied potential. Without producing byproducts, the WO_3_/BiVO_4_ photoanode completely degraded the S2NS in 60 min and BAC-C12 surfactants in 90 min. Direct photohole oxidation was found to be responsible for surfactant degradation. Compared to cationic-type surfactants, the WO_3_/BiVO_4_ photoanode was favorable for anionic surfactant degradation due to its higher affinity for photogenerated holes. Photocurrent generation as well as hydrogen evolution was independent of the surfactant present in the electrolyte. The coproduction of H_2_O_2_ opens up possibilities of effectively treating real surfactant wastewater containing other contaminants where direct photohole oxidation of surfactants can occur in conjunction with H_2_O_2_ based degradation of coexisting pollutants. This work demonstrates the capability of an effective water-energy nexus, and therefore forms the basis for coupling wastewater treatment and energy generation required for an accelerated transition toward net zero.

## Supporting Information Available

Detail for photoanode fabrication, SEM images of the photoanode, composition analysis, optical properties, and GC-MS results of surfactant degradation (PDF)

## Abbreviations

AOP: Advanced oxidation processes
BAC-C12: Benzyl alkyl dimethylammonium compounds
CID: Collision induced dissociation
DRS: Diffuse reflectance spectra
FTO: Fluorine-doped tin oxide
GC: Gas chromatography
HAADF: High-angle annular dark-field imaging
HPLC: High-performance liquid chromatography
HRTEM: High resolution-transmission electron microscopy
LAS: Linear alkylbenzenesulfonates
LC: Liquid chromatography
PEC: Photoelectrocatalysis
QACs: Quaternary ammonium compounds
RHE: Reversible hydrogen electrode
S2NS: Sodium 2-naphthalenesulfonate
SEM: Scanning electron microscopy
XPS: X-photoelectron spectroscopy
XRD: X-ray diffraction

## References

[ref1] Nakama Y. Chapter 15 - Surfactants. In Cosmetic Science and Technology; Sakamoto K., Lochhead R. Y., Maibach H. I., Yamashita Y., Eds.; Elsevier: Amsterdam, 2017; pp 231–244.

[ref2] Ivanković T.; Hrenović J. Surfactants in the environment. Arh Hig Rada Toksikol 2010, 61 (1), 95–110. 10.2478/10004-1254-61-2010-1943. 20338873

[ref3] Mukherjee A.; Mullick A.; Vadthya P.; Moulik S.; Roy A. Surfactant degradation using hydrodynamic cavitation based hybrid advanced oxidation technology: A techno economic feasibility study. Chemical Engineering Journal 2020, 398, 125599 10.1016/j.cej.2020.125599.

[ref4] Ying G.-G. Fate, behavior and effects of surfactants and their degradation products in the environment. Environ. Int. 2006, 32 (3), 417–431. 10.1016/j.envint.2005.07.004. 16125241

[ref5] Wang Y.; Zhang Y.; Li X.; Sun M.; Wei Z.; Wang Y.; Gao A.; Chen D.; Zhao X.; Feng X. Exploring the Effects of Different Types of Surfactants on Zebrafish Embryos and Larvae. Sci. Rep. 2015, 5 (1), 10107 10.1038/srep10107. 26053337 PMC4459078

[ref6] Nunes R. F.; Teixeira A. C. S. C. An overview on surfactants as pollutants of concern: Occurrence, impacts and persulfate-based remediation technologies. Chemosphere 2022, 300, 134507 10.1016/j.chemosphere.2022.134507. 35395256

[ref7] Hora P. I.; Arnold W. A. Photochemical fate of quaternary ammonium compounds in river water. Environmental Science: Processes & Impacts 2020, 22 (6), 1368–1381. 10.1039/D0EM00086H. 32406464

[ref8] Fernández P.; Alder A. C.; Suter M. J. F.; Giger W. Determination of the Quaternary Ammonium Surfactant Ditallowdimethylammonium in Digested Sludges and Marine Sediments by Supercritical Fluid Extraction and Liquid Chromatography with Postcolumn Ion-Pair Formation. Anal. Chem. 1996, 68 (5), 921–929. 10.1021/ac9505482. 21619191

[ref9] Martínez-Carballo E.; Sitka A.; González-Barreiro C.; Kreuzinger N.; Fürhacker M.; Scharf S.; Gans O. Determination of selected quaternary ammonium compounds by liquid chromatography with mass spectrometry. Part I. Application to surface, waste and indirect discharge water samples in Austria. Environ. Pollut. 2007, 145 (2), 489–496. 10.1016/j.envpol.2006.04.033. 16835005

[ref10] Sakai N.; Shirasaka J.; Matsui Y.; Ramli M. R.; Yoshida K.; Ali Mohd M.; Yoneda M. Occurrence, fate and environmental risk of linear alkylbenzene sulfonate in the Langat and Selangor River basins, Malaysia. Chemosphere 2017, 172, 234–241. 10.1016/j.chemosphere.2016.12.139. 28081507

[ref11] da Silva Coelho K.; Rocha O. Assessment of the potential toxicity of a linear alkylbenzene sulfonate (LAS) to freshwater animal life by means of cladoceran bioassays. Ecotoxicology 2010, 19 (4), 812–818. 10.1007/s10646-009-0458-3. 20091119

[ref12] Rao N. N.; Dube S. Photocatalytic degradation of mixed surfactants and some commercial soap/detergent products using suspended TiO2 catalysts. J. Mol. Catal. A: Chem. 1996, 104 (3), L197–L199. 10.1016/1381-1169(95)00259-6.

[ref13] Hidaka H.; Zhao J.; Pelizzetti E.; Serpone N. Photodegradation of surfactants. 8. Comparison of photocatalytic processes between anionic DBS and cationic BDDAC on the titania surface. J. Phys. Chem. 1992, 96 (5), 2226–2230. 10.1021/j100184a037.

[ref14] Kuźmiński K.; Morawski A. W.; Janus M. Adsorption and Photocatalytic Degradation of Anionic and Cationic Surfactants on Nitrogen-Modified TiO2. J. Surfactants Deterg. 2018, 21 (6), 909–921. 10.1002/jsde.12190.

[ref15] Nguyen H. M.; Phan C. M.; Sen T.; Hoang S. A. TOC removal from laundry wastewater by photoelectrochemical process on Fe2O3 nanostructure. Desalination and Water Treatment 2016, 57 (31), 14379–14385. 10.1080/19443994.2015.1064036.

[ref16] Nguyen H. M.; Phan C. M.; Sen T. Degradation of sodium dodecyl sulfate by photoelectrochemical and electrochemical processes. Chemical Engineering Journal 2016, 287, 633–639. 10.1016/j.cej.2015.11.074.

[ref17] Paschoal F. M. M.; Anderson M. A.; Zanoni M. V. B. Photoeletrocatalytic oxidation of anionic surfactant used in leather industry on nanoporous Ti/TiO2 eletrodes. J. Braz. Chem. Soc. 2008, 19, 803 10.1590/S0103-50532008000400027.

[ref18] Ahmari H.; Zeinali Heris S.; Hassanzadeh Khayyat M. Photo catalytic degradation of linear alkylbenzene sulfonic acid. Res. Chem. Intermed. 2016, 42 (8), 6587–6606. 10.1007/s11164-016-2483-1.

[ref19] Pitchaimuthu S.; Sridharan K.; Nagarajan S.; Ananthraj S.; Robertson P.; Kuehnel M. F.; Irabien A.; Maroto-Valer M. Solar Hydrogen Fuel Generation from Wastewater–Beyond Photoelectrochemical Water Splitting: A Perspective. Energies 2022, 15 (19), 7399 10.3390/en15197399.

[ref20] Jones B.; Davies K. R.; Allan M. G.; Anantharaj S.; Mabbett I.; Watson T.; Durrant J. R.; Kuehnel M. F.; Pitchaimuthu S. Photoelectrochemical concurrent hydrogen generation and heavy metal recovery from polluted acidic mine water. Sustainable Energy & Fuels 2021, 5 (12), 3084–3091. 10.1039/D1SE00232E.

[ref21] Kurnia F.; Scott J. A.; Valanoor N.; Hart J. N. A review of non-oxide semiconductors for photoelectrochemical water splitting. Journal of Materials Chemistry C 2023, 11 (3), 802–826. 10.1039/D2TC02533G.

[ref22] Li S.; Xu W.; Meng L.; Tian W.; Li L. Recent Progress on Semiconductor Heterojunction-Based Photoanodes for Photoelectrochemical Water Splitting. Small Science 2022, 2 (5), 2100112 10.1002/smsc.202100112. PMC11935891 40212603

[ref23] Choi J.; Song T.; Kwon J.; Lee S.; Han H.; Roy N.; Terashima C.; Fujishima A.; Paik U.; Pitchaimuthu S. WO3 nanofibrous backbone scaffolds for enhanced optical absorbance and charge transport in metal oxide (Fe2O3, BiVO4) semiconductor photoanodes towards solar fuel generation. Appl. Surf. Sci. 2018, 447, 331–337. 10.1016/j.apsusc.2018.03.167.

[ref24] Zou X.; Sun Z.; Hu Y. H. g-C3N4-based photoelectrodes for photoelectrochemical water splitting: a review. Journal of Materials Chemistry A 2020, 8 (41), 21474–21502. 10.1039/D0TA07345H.

[ref25] Nandjou F.; Haussener S. Kinetic Competition between Water-Splitting and Photocorrosion Reactions in Photoelectrochemical Devices. ChemSusChem 2019, 12 (9), 1984–1994. 10.1002/cssc.201802558. 30644167

[ref26] Guo L.-J.; Luo J.-W.; He T.; Wei S.-H.; Li S.-S. Photocorrosion-Limited Maximum Efficiency of Solar Photoelectrochemical Water Splitting. Physical Review Applied 2018, 10 (6), 064059 10.1103/PhysRevApplied.10.064059.

[ref27] Xiong Z.; Hu C.; Luo X.; Zhou W.; Jiang Z.; Yang Y.; Yu T.; Lei W.; Yuan C. Field-Free Improvement of Oxygen Evolution Reaction in Magnetic Two-Dimensional Heterostructures. Nano Lett. 2021, 21 (24), 10486–10493. 10.1021/acs.nanolett.1c03981. 34859672

[ref28] Peng D.; Hu C.; Luo X.; Huang J.; Ding Y.; Zhou W.; Zhou H.; Yang Y.; Yu T.; Lei W.; Yuan C. Electrochemical Reconstruction of NiFe/NiFeOOH Superparamagnetic Core/Catalytic Shell Heterostructure for Magnetic Heating Enhancement of Oxygen Evolution Reaction. Small 2023, 19 (3), 2205665 10.1002/smll.202205665. 36404111

[ref29] Liu Z.; Jiang Z.; Luo X.; Zhou W.; Chen M.; Su M.; Shi P.; Hou Y.; Xiong Z.; Li Q.; Yu T.; Yuan C. Prolonging lifetime of photogenerated carriers in WO3 nanowires by oxygen vacancies engineering for enhanced photoelectrocatalytic oxygen evolution reaction. Appl. Phys. Lett. 2021, 119 (10), 103901 10.1063/5.0061973.

[ref30] Gong X.; Jiang Z.; Zeng W.; Hu C.; Luo X.; Lei W.; Yuan C. Alternating Magnetic Field Induced Magnetic Heating in Ferromagnetic Cobalt Single-Atom Catalysts for Efficient Oxygen Evolution Reaction. Nano Lett. 2022, 22 (23), 9411–9417. 10.1021/acs.nanolett.2c03359. 36410739

[ref31] Choi J.; Sudhagar P.; Kim J. H.; Kwon J.; Kim J.; Terashima C.; Fujishima A.; Song T.; Paik U. WO3/W:BiVO4/BiVO4 graded photoabsorber electrode for enhanced photoelectrocatalytic solar light driven water oxidation. Phys. Chem. Chem. Phys. 2017, 19 (6), 4648–4655. 10.1039/C6CP08199A. 28124693

[ref32] Schanz T.; Burek B. O.; Bloh J. Z. Fate and Reactivity of Peroxides Formed over BiVO4 Anodes in Bicarbonate Electrolytes. ACS Energy Letters 2023, 8 (3), 1463–1467. 10.1021/acsenergylett.3c00227.

[ref33] Fuku K.; Miyase Y.; Miseki Y.; Funaki T.; Gunji T.; Sayama K. Photoelectrochemical Hydrogen Peroxide Production from Water on a WO3/BiVO4 Photoanode and from O2 on an Au Cathode Without External Bias. Chem. Asian J. 2017, 12 (10), 1111–1119. 10.1002/asia.201700292. 28332317

[ref34] Fuku K.; Sayama K. Efficient oxidative hydrogen peroxide production and accumulation in photoelectrochemical water splitting using a tungsten trioxide/bismuth vanadate photoanode. Chem. Commun. 2016, 52 (31), 5406–9. 10.1039/C6CC01605G. 27009778

[ref35] Zhang K.; Liu J.; Wang L.; Jin B.; Yang X.; Zhang S.; Park J. H. Near-Complete Suppression of Oxygen Evolution for Photoelectrochemical H2O Oxidative H2O2 Synthesis. J. Am. Chem. Soc. 2020, 142 (19), 8641–8648. 10.1021/jacs.9b13410. 32160742

[ref36] Kafizas A.; Xing X.; Selim S.; Mesa C. A.; Ma Y.; Burgess C.; McLachlan M. A.; Durrant J. R. Ultra-thin Al2O3 coatings on BiVO4 photoanodes: Impact on performance and charge carrier dynamics. Catal. Today 2019, 321–322, 59–66. 10.1016/j.cattod.2017.11.014.

[ref37] Brack P.; Sagu J. S.; Peiris T. A. N.; McInnes A.; Senili M.; Wijayantha K. G. U.; Marken F.; Selli E. Aerosol-Assisted CVD of Bismuth Vanadate Thin Films and Their Photoelectrochemical Properties. Chem. Vap. Deposition 2015, 21 (1–2-3), 41–45. 10.1002/cvde.201407142.

[ref38] Grigioni I.; Di Liberto G.; Dozzi M. V.; Tosoni S.; Pacchioni G.; Selli E. WO3/BiVO4 Photoanodes: Facets Matching at the Heterojunction and BiVO4 Layer Thickness Effects. ACS Applied Energy Materials 2021, 4 (8), 8421–8431. 10.1021/acsaem.1c01623. 34485843 PMC8414527

[ref39] Zhang X.; Wang X.; Wang D.; Ye J. Conformal BiVO4-Layer/WO3-Nanoplate-Array Heterojunction Photoanode Modified with Cobalt Phosphate Cocatalyst for Significantly Enhanced Photoelectrochemical Performances. ACS Appl. Mater. Interfaces 2019, 11 (6), 5623–5631. 10.1021/acsami.8b05477. 30004671

[ref40] Wan W.; Su J.; Zou X. D.; Willhammar T. Transmission electron microscopy as an important tool for characterization of zeolite structures. Inorganic Chemistry Frontiers 2018, 5 (11), 2836–2855. 10.1039/C8QI00806J.

[ref41] Zhou W.; Greer H. F. What Can Electron Microscopy Tell Us Beyond Crystal Structures?. Eur. J. Inorg. Chem. 2016, 2016 (7), 941–950. 10.1002/ejic.201501342.

[ref42] Nareejun W.; Ponchio C. Novel photoelectrocatalytic/solar cell improvement for organic dye degradation based on simple dip coating WO3/BiVO4 photoanode electrode. Sol. Energy Mater. Sol. Cells 2020, 212, 110556 10.1016/j.solmat.2020.110556.

[ref43] Chatchai P.; Murakami Y.; Kishioka S.-y.; Nosaka A. Y.; Nosaka Y. Efficient photocatalytic activity of water oxidation over WO3/BiVO4 composite under visible light irradiation. Electrochim. Acta 2009, 54 (3), 1147–1152. 10.1016/j.electacta.2008.08.058.

[ref44] Darmawi S.; Burkhardt S.; Leichtweiss T.; Weber D. A.; Wenzel S.; Janek J.; Elm M. T.; Klar P. J. Correlation of electrochromic properties and oxidation states in nanocrystalline tungsten trioxide. Phys. Chem. Chem. Phys. 2015, 17 (24), 15903–15911. 10.1039/C5CP02482J. 26018838

[ref45] Mali S. S.; Park G. R.; Kim H.; Kim H. H.; Patil J. V.; Hong C. K. Synthesis of nanoporous Mo:BiVO4 thin film photoanodes using the ultrasonic spray technique for visible-light water splitting. Nanoscale Advances 2019, 1 (2), 799–806. 10.1039/C8NA00209F. 36132239 PMC9473260

[ref46] Cui T.; Su Y.; Fu X.; Zhu Y.; Zhang Y. The key role of surface hydroxyls on the activity and selectivity in photocatalytic degradation of organic pollutants and NO removal. J. Alloys Compd. 2022, 921, 165931 10.1016/j.jallcom.2022.165931.

[ref47] Zeng Q.; Li J.; Li L.; Bai J.; Xia L.; Zhou B. Synthesis of WO3/BiVO4 photoanode using a reaction of bismuth nitrate with peroxovanadate on WO3 film for efficient photoelectrocatalytic water splitting and organic pollutant degradation. Applied Catalysis B: Environmental 2017, 217, 21–29. 10.1016/j.apcatb.2017.05.072.

[ref48] Sudhagar P.; Song T.; Devadoss A.; Lee J. W.; Haro M.; Terashima C.; Lysak V. V.; Bisquert J.; Fujishima A.; Gimenez S.; Paik U. Modulating the interaction between gold and TiO2 nanowires for enhanced solar driven photoelectrocatalytic hydrogen generation. Phys. Chem. Chem. Phys. 2015, 17 (29), 19371–19378. 10.1039/C5CP01175B. 26143888

[ref49] Zhang S.; Ahmet I.; Kim S.-H.; Kasian O.; Mingers A. M.; Schnell P.; Kölbach M.; Lim J.; Fischer A.; Mayrhofer K. J. J.; Cherevko S.; Gault B.; van de Krol R.; Scheu C. Different Photostability of BiVO4 in Near-pH-Neutral Electrolytes. ACS Applied Energy Materials 2020, 3 (10), 9523–9527. 10.1021/acsaem.0c01904. 33134878 PMC7592387

[ref50] Gao R.-T.; Wang L. Stable Cocatalyst-Free BiVO4 Photoanodes with Passivated Surface States for Photocorrosion Inhibition. Angew. Chem., Int. Ed. 2020, 59 (51), 23094–23099. 10.1002/anie.202010908. 32888248

[ref51] Zhang Y.; Xiong X.; Han Y.; Zhang X.; Shen F.; Deng S.; Xiao H.; Yang X.; Yang G.; Peng H. Photoelectrocatalytic degradation of recalcitrant organic pollutants using TiO2 film electrodes: An overview. Chemosphere 2012, 88 (2), 145–154. 10.1016/j.chemosphere.2012.03.020. 22483728

[ref52] Garcia-Segura S.; Brillas E. Applied photoelectrocatalysis on the degradation of organic pollutants in wastewaters. Journal of Photochemistry and Photobiology C: Photochemistry Reviews 2017, 31, 1–35. 10.1016/j.jphotochemrev.2017.01.005.

[ref53] Yang J.; Dai J.; Chen C.; Zhao J. Effects of hydroxyl radicals and oxygen species on the 4-chlorophenol degradation by photoelectrocatalytic reactions with TiO2-film electrodes. J. Photochem. Photobiol., A 2009, 208 (1), 66–77. 10.1016/j.jphotochem.2009.08.007.

[ref54] Zhou Y.; Zhang G.; Zou J. Photoelectrocatalytic generation of miscellaneous oxygen-based radicals towards cooperative degradation of multiple organic pollutants in water. Journal of Water Reuse and Desalination 2021, 11, 531 10.2166/wrd.2021.018.

[ref55] Serpone N.; Emeline A. V. Suggested terms and definitions in photocatalysis and radiocatalysis. International Journal of Photoenergy 2002, 4, 91 10.1155/S1110662X02000144.

[ref56] Yang M.; He H.; Du J.; Peng H.; Ke G.; Zhou Y. Insight into the Kinetic Influence of Oxygen Vacancies on the WO3 Photoanodes for Solar Water Oxidation. J. Phys. Chem. Lett. 2019, 10 (20), 6159–6165. 10.1021/acs.jpclett.9b02365. 31552737

[ref57] Papagiannis I.; Stathi P.; Deligiannakis Y.; Keramidas A.; Lianos P. Photoelectrocatalytic production of hydrogen peroxide using a photo(catalytic) fuel cell. J. Photochem. Photobiol., A 2020, 389, 112210 10.1016/j.jphotochem.2019.112210.

[ref58] Zanoni M. V. B.; Sene J. J.; Selcuk H.; Anderson M. A. Photoelectrocatalytic Production of Active Chlorine on Nanocrystalline Titanium Dioxide Thin-Film Electrodes. Environ. Sci. Technol. 2004, 38 (11), 3203–3208. 10.1021/es0347080. 15224756

[ref59] Wu X.; Huang Z.; Liu Y.; Fang M. Investigation on the Photoelectrocatalytic Activity of Well-Aligned TiO_**2**_ Nanotube Arrays. International Journal of Photoenergy 2012, 2012, 832516 10.1155/2012/832516.

[ref60] Gu C.; Wang J.; Zhao Z.; Han Y.; Du M.; Zan S.; Wang F. Aerobic cometabolism of tetrabromobisphenol A by marine bacterial consortia. Environmental Science and Pollution Research 2019, 26, 23832–23841. 10.1007/s11356-019-05660-7. 31209756

