# Supplementary material for: Photoelectrocatalytic Surfactant Pollutant Degradation and Simultaneous Green Hydrogen Generation
Source: Ind Eng Chem Res. 2023 Jun 2;62(45):19084–94. doi: 10.1021/acs.iecr.3c00840 (PMC10655085; doi:10.1021/acs.iecr.3c00840)
Supplement: Supplementary file 1 — ie3c00840_si_001.pdf [file ie3c00840_si_001.pdf]

Supporting Information

Photoelectrocatalytic Surfactant Pollutant Degradation and Simultaneous Green Hydrogen Generation

Katherine Rebecca Davies,[a] Michael G. Allan,[b] Sanjay Nagarajan,[c] Rachel Townsend,[d] Vijayshankar Asokan,[e] Trystan Watson,[a] A. Ruth Godfrey,[d] M. Mercedes Maroto-Valer,[f] Moritz F. Kuehnel,[b,g] Sudhagar Pitchaimuthu[a,f*]

a. SPECIFIC, Faculty of Science and Engineering, Swansea University, Swansea, Wales.

b. Department of Chemistry, Faculty of Science and Engineering, Swansea University, Singleton Park, SA2 8PP, Swansea, Wales.

c. Department of Chemical Engineering, University of Bath, Bath BA2 7AY, UK.

d. Swansea University Medical School, Singleton Park, Swansea, SA2 8PP, UK

e. Energy and Materials, Chalmers University of Technology, Kemivägen 10, 41296 Gothenburg, Sweden.

f. Research Centre for Carbon Solutions, Institute of Mechanical, Processing and Energy Engineering, School of Engineering and Physical Sciences, Heriot-Watt University, Edinburgh, UK

g. Fraunhofer Institute for Wind Energy Systems IWES, Am Hauptor 4310, 06237 Leuna, Germany

Corresponding Author

S.Pitchaimuthu@hw.ac.uk (SP)

S1. Experimental Details:

Preparation of mesoporous $WO_3/BiVO_4$ Photoanode

In the first step, a homogenous WO_3 paste was prepared using commercial WO_3 nanocrystalline powder (100 nm size). We adopted Regan's protocol where WO_3 nanopowder was utilised instead of TiO_2[1]. Briefly, the preparation involved the following stages, (a) grinding 5 g of WO_3 with 1 mL of acetic acid for 5 minutes using mortar and pestle, (b) adding 1 mL of deionised water and grinding for 1 minute (repeat this 6 times), (c) adding 1 mL of ethanol and grinding for 1 minute (repeat this 15 times), (d) adding 2.5 mL of ethanol and grinding for 1 minute (repeat 6 times). Then, the WO_3 colloid was transferred into a closed beaker using 100 mL of ethanol. The solution was then magnetically stirred (150 RPM) for 1 minute, sonicated for 10 minutes (10 seconds on/off) and magnetically stirred (150 RPM) for 1 minute again. Then 20 g of α-terpineol was added to the above colloidal solution, and then it was magnetically stirred for 1 minute, sonicated for 10 minutes (10 seconds on/off) and magnetically stirred for 1 minute again. Once this step was completed, then 30g of 10% (w/w) ethyl cellulose in ethanol was added, and the solution was magnetically stirred for 1 minute, sonicated for 10 minutes (10 seconds on/off), and magnetically stirred for 1 minute again. The final step used a rotary evaporator to remove the ethanol from the paste. The setting was under vacuum, and the temperature ranged from 50 – 70 °C, the speed of rotation was 90 – 120 RPM, and it was conducted for 1 hour. Once the homogenous WO_3 paste was ready, it was applied on the pre-cleaned fluorine-doped tin oxide coated (FTO) glass (Pilkington 12 Ω sheet resistance) by utilising the doctor blade method. A hotplate was utilised to anneal the samples by utilising the following conditions: (a) Ramp Time = 1 hour, (b) Set temperature = 450 °C and (c) Annealing Time = 3 hours. This step was repeated twice to make a 2-layer WO_3 on the FTO glass.

The $BiVO_4$ solution was prepared by utilising the method reported by Choi et al. [2]. This was done by mixing 0.1462 g of ammonium metavanadate, 0.6061 g of bismuth

nitrate pentahydrate, 0.4803 g of citric acid, 0.825 g of nitric acid and 2.9 mL of deionised water together. The solution was then sonicated for 10 minutes. Once the solution was finished, it was spin-coated on top of the WO_3 surface by utilising a spin coater with a setting of 500 RPM for 5 seconds and 2000 RPM for 40 seconds. The $WO_3/BiVO_4$ photoanode was annealed using the following settings on an enclosed hotplate: (a) Ramp Time = 2.5 hours, (b) Set Temperature = 500 °C and (c) Annealing Time = 1 hour. Finally, an insulated copper wire was attached to the uncoated area of $WO_3/BiVO_4$ coating on FTO glass, and the electrode was insulated to avoid the electrolyte interaction using epoxy resin glue. The active area of the electrode was approximately 1 cm^2.

S2. X-ray diffraction results

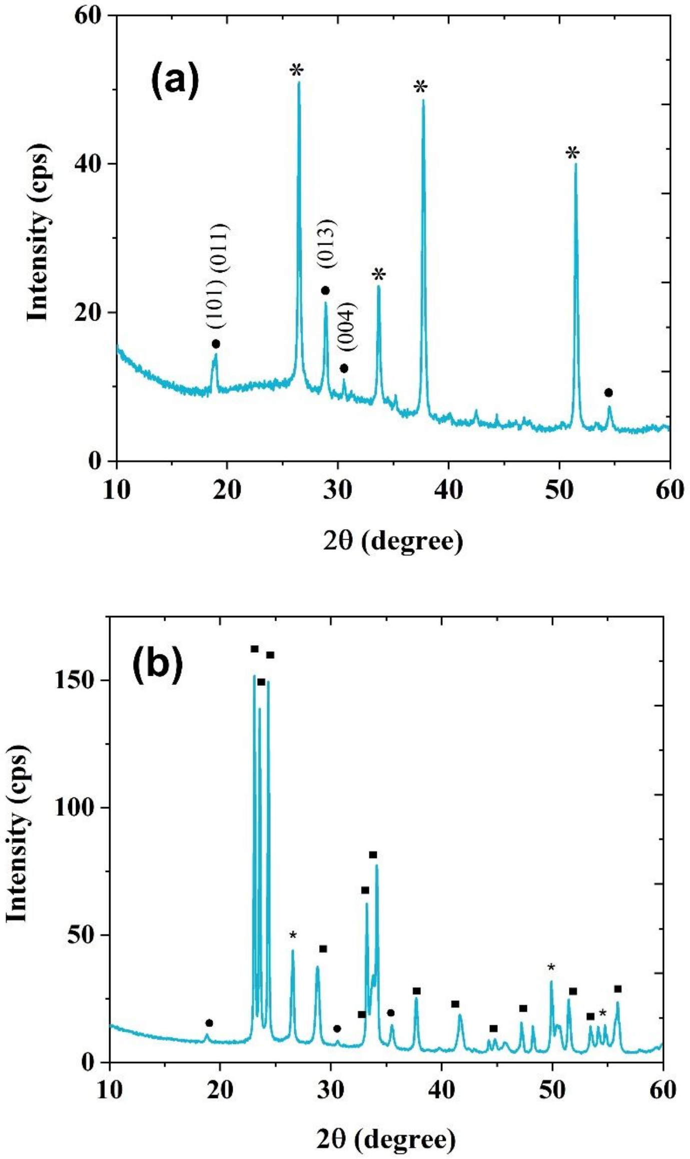

Figure S1. XRD results of (a) $BiVO_4$ and (b) WO_3/BiVO4 thin films coated onto FTO substrate. The ● symbol represents $BiVO_4$ crystallite peaks, * symbol indicates the FTO substrate peaks. The rectangular symbols showed the WO_3 crystallite peaks.

From Figure S1 a the peaks exhibits around 18.9 °and 28.9° peaks correspond to the (011) and (013) reflections of monoclinic scheelite BiVO4 (PDF 01–075-1866)[3]. The other predominant peaks are attributed to FTO substrate. When $BiVO_4$ film coated onto WO_3 films, it exhibits a weaker crystallite peaks due to high crystalline nature of WO_3 film.

S3. Surface morphology analysis

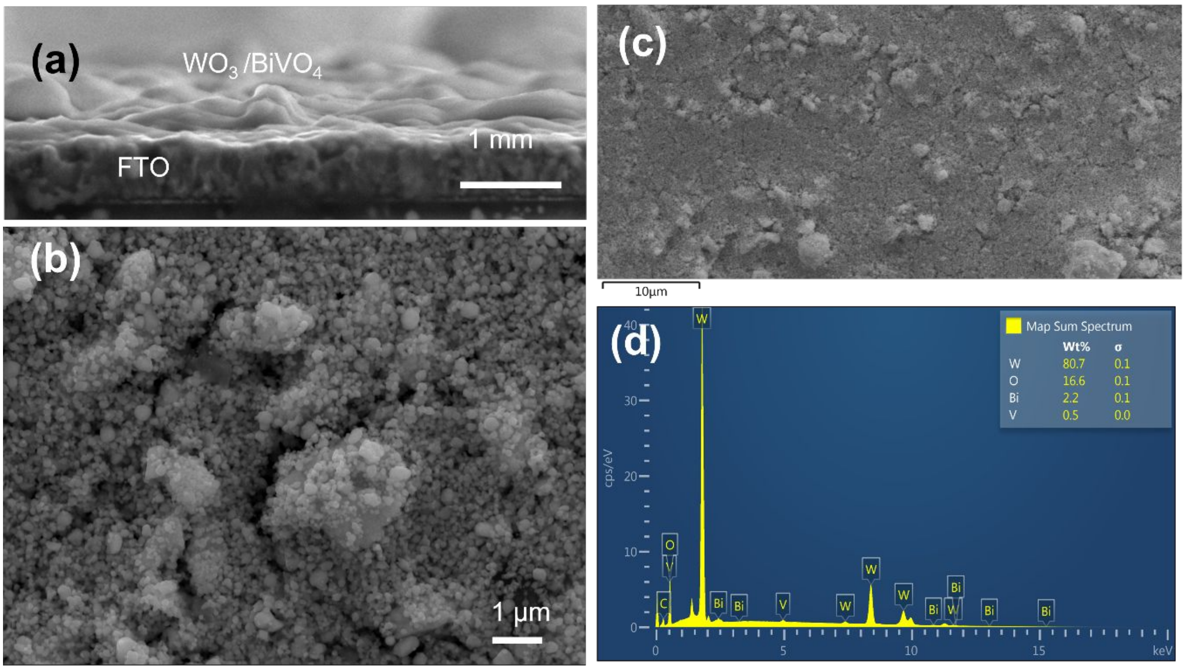

Figure S2. (a) Cross section SEM image of $WO_3/BiVO_4$ thin film coated onto FTO substrate. Planar SEM image of $WO_3/BiVO_4$ film at (b) 1 µm and (c) 10 µm scale, and (d) elemental analysis recorded along Figure S2(b).

Table S1. Elemental composition (Wt%) of $WO_3/BiVO_4$ photoanode before and after PEC reactions.

Elements	Before PEC reaction Wt (%)	After PEC reaction Wt (%)
W	80.7	79.1
O	16.6	18.3
Bi	2.2	2.2
Vi	0.5	0.2

S4. Optical analysis

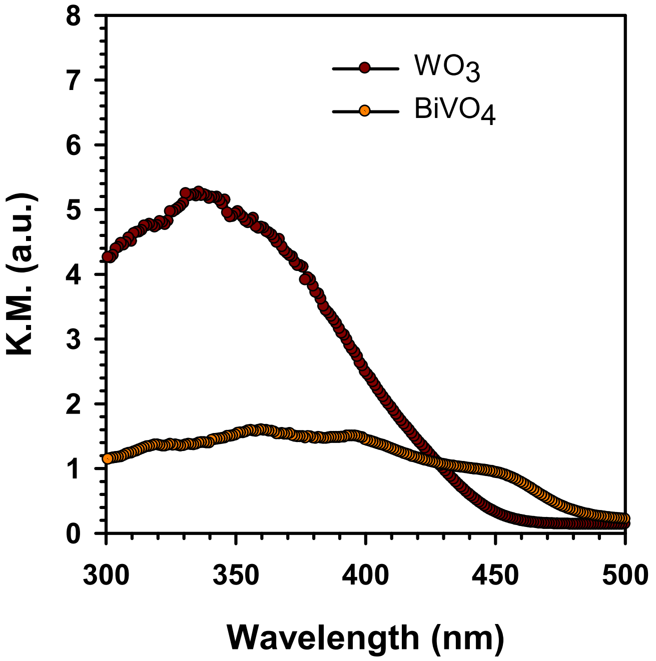

Figure S3. Diffused reflectance spectra of WO_3 and $BiVO_4$ coated films on FTO substrate.

S5. Liquid chromatography results

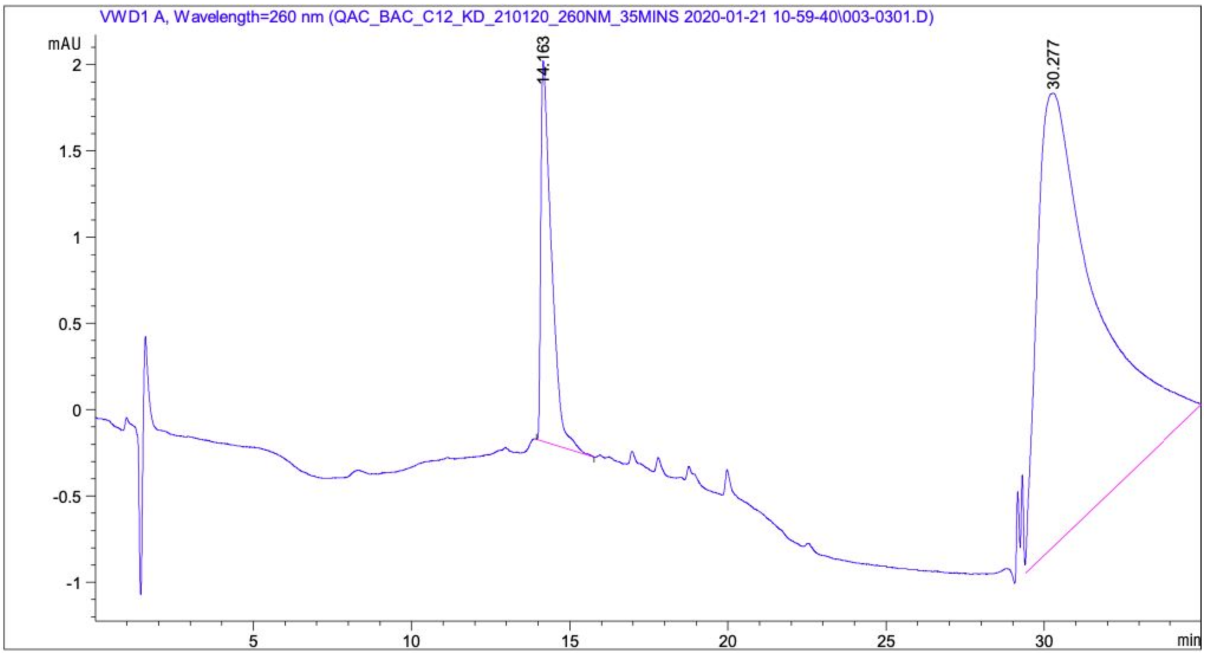

Figure S4 The chromatogram obtained for a pure BAC-C12 standard after benchmarking.

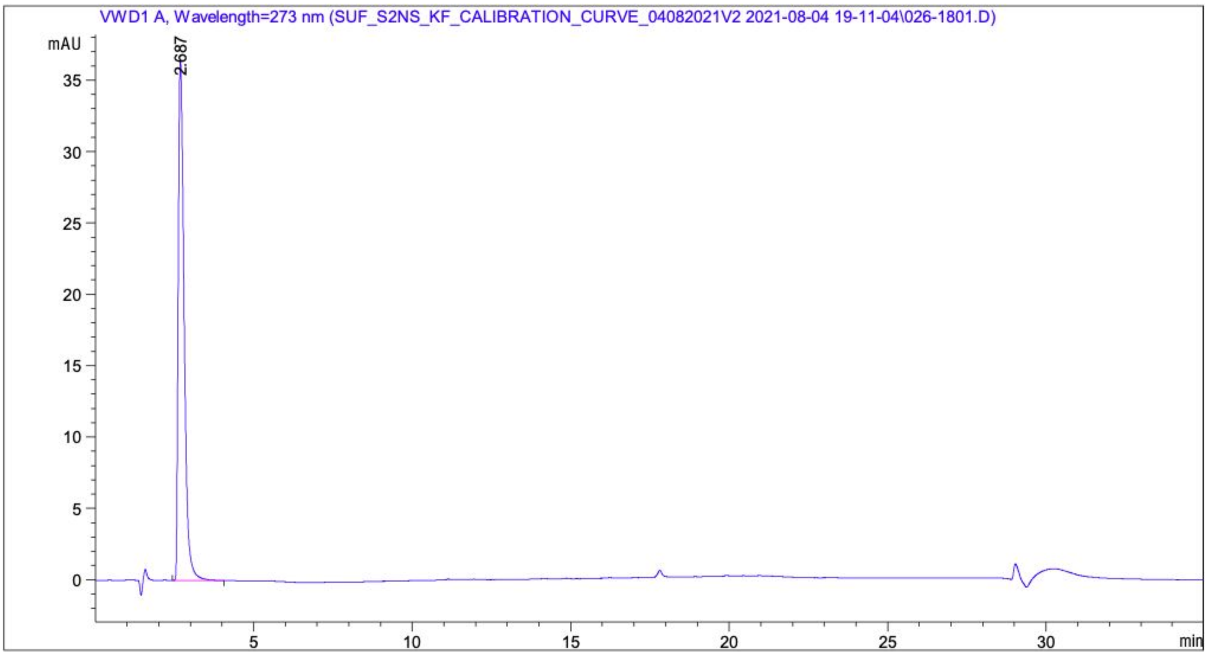

Figure S5. The chromatogram obtained for a pure S2NS standard after benchmarking.

S6. Surfactants adsorption at dark

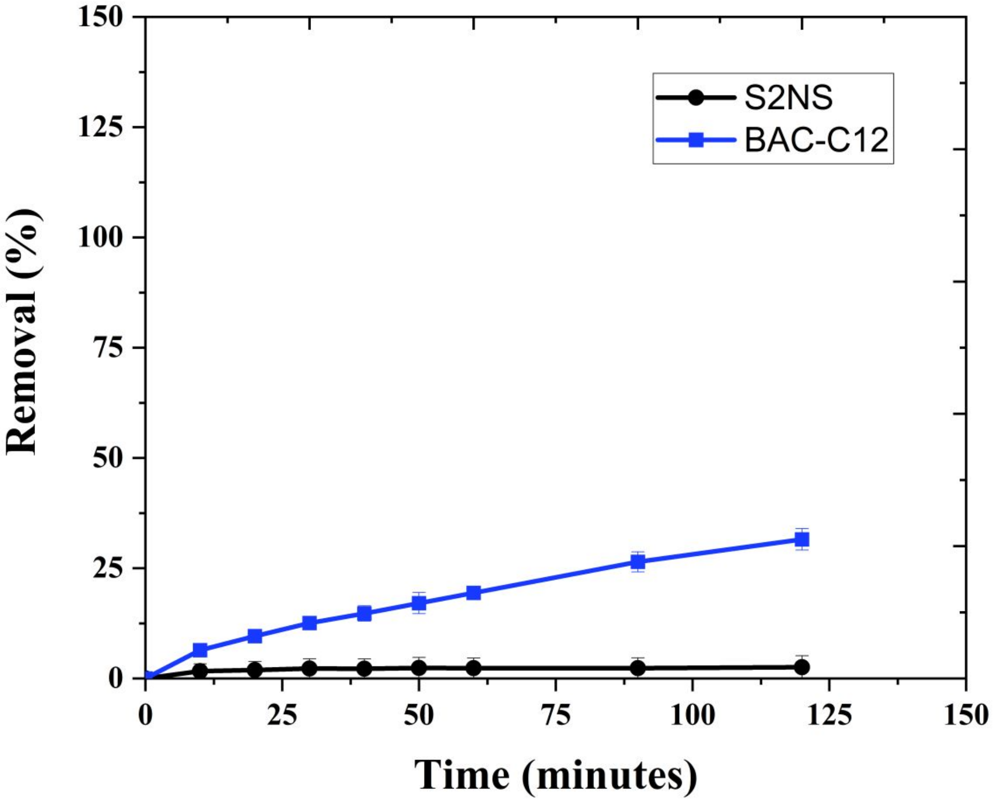

Figure S6. Surfactants removal (%) via surface adsorption on photoanode at dark.

S7. Influence of surfactants initial concentration

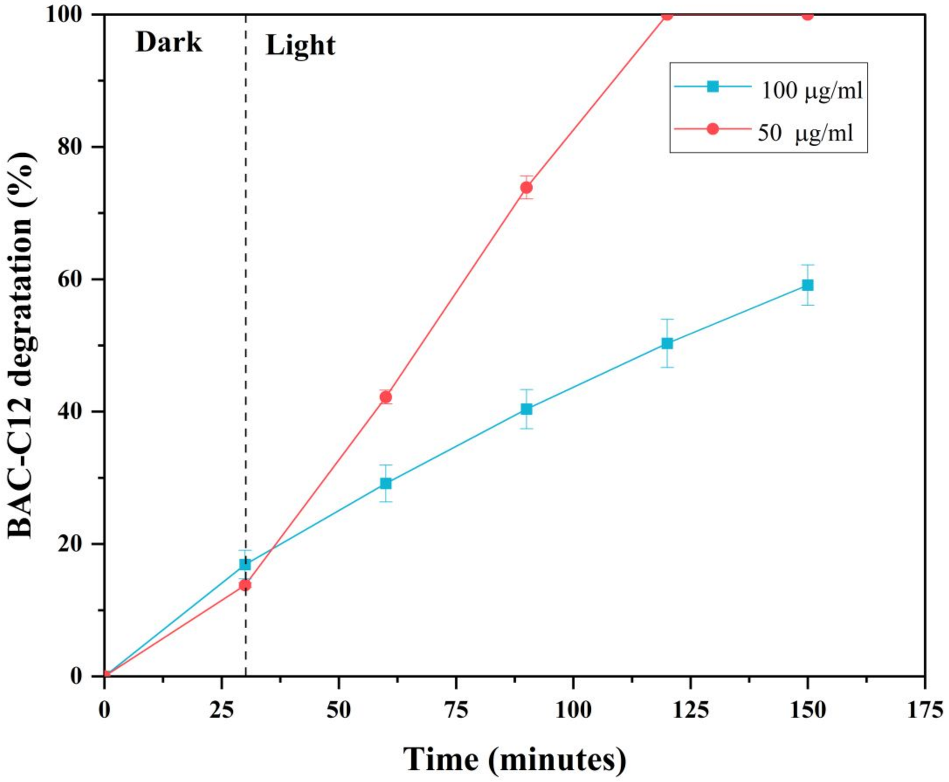

Figure S7. PEC surfactant degradation (%) of BAC-C12 at dark and light irradiation conditions. The experiment was recorded at 1.75 V RHE applied potential.

References

1. O'Regan, B.; Grätzel, M., A low-cost, high-efficiency solar cell based on dye-sensitized colloidal TiO2 films. *Nature* **1991,** 353, (6346), 737-740.

2. Choi, J.; Sudhagar, P.; Kim, J. H.; Kwon, J.; Kim, J.; Terashima, C.; Fujishima, A.; Song, T.; Paik, U., WO3/W:BiVO4/BiVO4 graded photoabsorber electrode for enhanced photoelectrocatalytic solar light driven water oxidation. *Physical Chemistry Chemical Physics* **2017,** 19, (6), 4648-4655.

3. Brack, P.; Sagu, J. S.; Peiris, T. A. N.; McInnes, A.; Senili, M.; Wijayantha, K. G. U.; Marken, F.; Selli, E., Aerosol-Assisted CVD of Bismuth Vanadate Thin Films and Their Photoelectrochemical Properties. *Chemical Vapor Deposition* **2015,** 21, (1-2-3), 41-45.
